# Where in the World Is This Research Taking Us? Collaborating on Publishable Research With Undergraduates Abroad

**DOI:** 10.3389/fpsyg.2019.00010

**Published:** 2019-01-25

**Authors:** Melissa Burns-Cusato, Brian Cusato

**Affiliations:** Department of Behavioral Neuroscience, Centre College, Danville, KY, United States

**Keywords:** research collaboration, undergraduate students, abroad, publishing, field study

The value of undergraduate student-faculty collaborative research has been well-documented in recent years (e.g., Hernandez et al., 2018), and as more colleges and universities recognize this, the models by which it is accomplished have become more varied. Some students engage in collaborative research with faculty at their home institutions while others spend the summer months researching at an institution other than their own. For students in the latter category, engaging in collaborative research away from one's home institution often exposes students to a greater variety of research questions and methodologies and facilitates the development of a professional network that often leads to important opportunities and future career success. In our research, we take this idea a step further by offering students the opportunity to conduct a research project in another country. This model of undergraduate collaboration aims to develop a more experienced, agile student researcher with a broader world view and a strong sense of global citizenship. In this paper we describe the benefits and challenges of collaborative research while abroad, through the lens of our own field research with students conducted over the past 10 years studying green monkeys on the Caribbean Island of Barbados.

## Benefits of Collaborative Research Abroad

Conducting collaborative research abroad presents both benefits and challenges to students and the faculty who mentor them. It has all of the characteristics associated with collaborative research in general, plus a host of additional attributes rooted in being immersed in a new and sometimes unfamiliar environment with new people, opportunities, expectations, and challenges that push students out of their comfort zone. For these and many other reasons, student-faculty collaborative research abroad has all of the advantages of study abroad—it enhances creativity (Godart et al., 2015), stimulates new ideas (Tadmor et al., 2012b), and improves communication skills (Marcotte et al., 2007). Moreover, study abroad has a lasting impact on student skills that directly impact their success in STEM-related fields—improved confidence and social competence (Walsh and Walsh, 2018), tolerance to ambiguity (Vande Berg et al., 2009), autonomy (Marcotte et al., 2007), critical thinking skills (Savicki et al., 2004), and cognitive flexibility (Tadmor et al., 2012a). In these ways and many more, experiences abroad are more likely to be transformative for students compared to traditional on-campus classes (Stone et al., 2017).

## General Considerations and Preparations For Faculty Mentors

Whether traveling alone or with students, one must weigh pros and cons and anticipate inherent challenges before taking on research in another country. There are considerable time lags to consider when acquiring travel documents (e.g., passports, travel visas), and many countries require special research permits and/or restrict the use of specialized research equipment. Moreover, dealing with two sets of bureaucratic requirements (e.g., U.S. and host country's IRB or IACUC) can be both time consuming and labor intensive. We recommend addressing these details, and others such as transportation, research site availability, and access to equipment and supplies as part of long-term planning. These tasks can be undertaken after arrival but anticipating and overcoming known challenges prior to departure maximizes productivity while abroad. Similarly, establishing a relationship with a researcher in the destination country is highly recommended. They will likely be more familiar with government offices, local requirements, and regulations regarding your planned activities.

## Preparing Student Researchers For Life Abroad

When taking undergraduate research assistants abroad, the research mentor assumes more responsibility for the students' well-being than would be the case when conducting research at one's home institution. Thus, it is essential that great care is taken, and more refined criteria employed, when selecting research assistants for travel. In addition to academic preparedness, consider the applicants' emotional stability (including their penchant for drama) and potential for engaging in risky behavior since mental health counselors and other campus support services are often unavailable while abroad. One must also consider health and well-being challenges associated with the host country. Prior to departure, review government websites like the one maintained by the US Department of State^1^ for current travel advisories. Vaccinations are required or recommended when visiting many foreign destinations. The Center for Disease Control Traveler's Health website^2^ provides a comprehensive list of vaccinations, other medical precautions to take prior to travel, and recommendations on how to avoid common medical problems while traveling in your host country. As an additional precaution, identify the best source of medical care near your lodging and study site before medical issues arise and share these details with your students, including all available transportation options. You should consider purchasing travel insurance for yourself and research assistants (some institutions provide this). This relatively inexpensive investment covers many unexpected expenses, including medical emergencies, that may occur while researching abroad.

The research mentor should also spend time prior to departure preparing research assistants for cultural differences they are likely to encounter while abroad. We have found that discussing cultural differences before students experience them facilitates their assimilation into the new environment. Common customs, traditions, and behavioral norms and expectations should be discussed, including culturally-appropriate dress. College-age students are more likely to take risks while traveling, and this can lead to “romantic encounters” with locals. Hence, it is best to educate your students about cultural differences in these interactions. For instance, polite discouragement may quell the advances of a suitor in one culture, but be interpreted as encouragement in another, resulting in a very uncomfortable misunderstanding, at best. Risk of sexual assault is much higher when traveling abroad (Kimble et al., 2013). Remind students of common sense safety measures instead of letting them surprise you with their naivety. Moreover, students tend to consume more alcohol while abroad compared to their drinking activity at home (Pedersen et al., 2010) which can potentially lead to alcohol-related problems (Hummer et al., 2010). Discussing dangerous drinking patterns can reduce alcohol use among college students (Cronce and Larimer, 2004) even during abroad experiences (Pedersen et al., 2017). Once safety and responsibility parameters are established, encourage students to embrace cultural differences and be open to new perspectives. Doing so will help students successfully navigate cultural nuances and better ensure a safe and successful research experience.

## Two Models For Collaborative Research Abroad

Once you have identified a good reason to conduct research in another country, there are two common models to consider. In one, the research mentor receives funding to support student collaborators and the research proceeds as a stand-alone project. In the other, research collaboration is embedded in a study abroad course led by the research mentor. Administrators looking to broaden study abroad opportunities are likely to view such a course very favorably since science and research courses abroad are relatively rare. With funding to support the project, a research mentor can focus efforts on the project and mentoring students through the research process. However, research budgets often provide support for only a small number of undergraduate assistants, thus limiting the amount of work that can be completed. In contrast, a study abroad course (ideally one focused on research methods) can be populated by capable students motivated to complete a research project for a grade. This is the model we have used with considerable success. In addition to traditional course requirements (e.g., assigned readings, presentations, exams), students in our Research in Primate Behavior course work in small groups on separate but related projects that are completed within a three-week winter or summer term. After the course has ended, we sometimes work at the study site with select research assistants for an another 2 weeks. Although not necessary, this time is used to run additional studies, or collect additional data related to the student projects that have promise for publication. We have found these models, alone, or in some combination, very useful when collaborating with students on publishable research abroad.

Regardless of whether one's research abroad follows the funded research project model or the course abroad model, likelihood of publication will be increased if the research team is able to complete multiple related studies during the time abroad. Conducting multiple studies simultaneously may be easiest to achieve by assigning each research assistant or group of students a different study to oversee. When preparing our students for their research abroad, we begin by introducing them to our over-arching research theme (e.g., anti-predator behavior in isolated green monkeys) and to our previous findings (Burns-Cusato et al., 2013, 2016). Special emphasis is placed on gaps in the literature and potential future directions. This process introduces important considerations, pointing students in appropriate directions without stifling creativity. We then give students the opportunity to develop ideas in brainstorming sessions. This strategy allows students to derive their own research questions, engendering intellectual ownership and personal investment in *their* projects, not projects we have hand-delivered. At the same time, we carefully mold these student-generated ideas into a cohesive collection of important (and feasible) research questions, based on our own knowledge and experience. While it may be counter to traditional scientific practices, we have students come up with their initial research ideas prior to reading the literature as a means of maximizing creativity. We use our familiarity with the literature to direct them away from unnecessary replications of prior work and ineffective research designs. Only after research questions have been vetted and first drafts of research designs have been determined do we provide students with a starter set of relevant literature. They are then required to do an extensive literature search on their specific project. After reading several related articles, we bring the students back together, and as a group, refine experimental designs and procedures.

Prior to arriving in the host country, students should be trained to properly execute experimental procedures. The mentor should then carefully oversee the start of every study, helping students hone data collection skills. As is often the case with student-faculty collaborations, early data may need to be discarded as you help students work toward accuracy and autonomy. When the mentor is confident in the students' abilities to follow the research protocol, students can collect data on their own during the day while nightly teaching efforts shift to data coding, analysis, and interpretation. During this phase, we have found it helpful to the success of student projects and hence, the likelihood of publication, to conduct research team meetings every evening. Here the research team reviews the progress of every project, including unexpected issues that occur during data collection or coding, how they may be addressed, and what preliminary analysis of the data has revealed. Examining the data during data collection is not typically a best practice, but when faced with time constraints, such analyses can reveal whether it is necessary to change methods or revise operational definitions before too much time is lost. Finally, we use these nightly meetings to review and discuss core concepts and emphasize the importance of every student's work to the overall research team and to the expansion of scientific knowledge.

## Final Thoughts

There are a few additional details to keep in mind before setting off on your research abroad adventure. First, carefully manage expectations for success. Unforeseen logistical challenges are likely, but overcoming these will provide invaluable knowledge in future excursions. Second, your research will not always go as planned. While this is true with domestic research, it is far more likely to be the case when you are working in an unfamiliar location and within a different culture. We encourage you to view these roadblocks as opportunities to step deeper into your host country's culture as you and your students interact with locals to help solve the problems you encounter. Overall, we are confident you will find research abroad a rewarding endeavor that expands student perspectives and skill sets, improves their ability to adapt to an ever-changing research landscape, and fosters ownership of their research projects. Ultimately, these outcomes will lead to success in publishing research findings.

## Author Contributions

MB-C and BC both contributed to the development of the theme and writing the manuscript.

### Conflict of Interest Statement

The authors declare that the research was conducted in the absence of any commercial or financial relationships that could be construed as a potential conflict of interest.

## Footnotes

^1^https://travel.state.gov/content/travel/en/traveladvisories/traveladvisories.html

^2^https://wwwnc.cdc.gov/travel/destinations/list

## References

[B1] Burns-Cusato M. Cusato B. Glueck A. C. (2013). Barbados green monkeys (Chlorocebus sabaeus) recognize ancestral alarm calls after 350 years of isolation. Behav. Processes 100, 197–199. 10.1016/j.beproc.2013.09.012 24129028

[B2] Burns-Cusato M. Glueck A. C. Merchak A. R. Palmer C. L. Rieskamp J. D. Duggan I. S.. (2016). Threats from the past: barbados green monkeys (Chlorocebus sabaeus) fear leopards after centuries of isolation. Behav. Processes 126, 1–11. 10.1016/j.beproc.2016.02.011 26910174

[B3] Cronce J. M. Larimer M. E. (2004). Individual-focused approaches to the prevention of college student drinking. Alcohol Res. Health. 34, 210–221. 22330220 PMC3342066

[B4] Godart F. C. Maddux W. W. Shipilov A. V. Galinsky A. D. (2015). Fashion with a foreign flair: professional experiences abroad facilitate the creative innovations of organizations. Acad. Manag. J. 58, 195–220. 10.5465/amj.2012.0575

[B5] Hernandez P. Woodcock A. Estrada M. Schultz P. W. (2018). Undergraduate research experiences broaden diversity in the scientific workforce. BioScience 68, 204–211. 10.1093/biosci/bix163

[B6] Hummer J. F. Pedersen E. R. Mirza T. LaBrie J. W. (2010). Factors associated with general and sexual alcohol-related consequences: an examination of college students while studying abroad. J. Student Affairs Res. Prac. 47, 427–444. 10.2202/1949-6605.6134 23505594 PMC3596165

[B7] Kimble M. Flack W. F. Burbridge E. (2013). Study abroad increases risk for sexual assault in female undergraduates: a preliminary report. Psychol. Trauma 5, 426–430. 10.1037/a0029608

[B8] Marcotte C. Desroches J. Poupart I. (2007). Preparing internationally minded business graduates: the role of international mobility programs. Int. J. Intercult. Relat. 31, 655–668. 10.1016/j.ijintrel.2007.05.002

[B9] Pedersen E. R. Larimer M. E. Lee C. M. (2010). When in Rome: factors associated with changes in drinking behavior among American college students studying abroad. Psychol. Addict. Behav. 24, 535–540. 10.1037/a0019863 20853940 PMC3576466

[B10] Pedersen E. R. Neighbors C. Atkins D. C. Lee C. M. Larimer M. E. (2017). Brief online interventions targeting risk and protective factors for increased and problematic alcohol use among american college students studying abroad. Psychol. Addict. Behav. 31, 220–230. 10.1037/adb0000242 28080092 PMC5344728

[B11] Savicki V. Downing-Burnette R. Heller L. Binder F. Suntinger W. (2004). Contrasts, changes, and correlates in actual and potential intercultural adjustment. Int. J. Intercult. Relat. 28, 311–329. 10.1016/j.ijintrel.2004.06.001

[B12] Stone G. A. Duerden M. D. Duffy L. N. Hill B. J. Witesman E. M. (2017). Measurement of transformative learning in study abroad: an application of the learning activities survey. J. Hospital, Leisure Sport Tourism Edu. 21, 23–32. 10.1016/j.jhlste.2017.05.003

[B13] Tadmor C. T. Galinsky A. D. Maddux W. W. (2012a). Getting the most out of living abroad: biculturalism and integrative complexity as key drivers of creative and professional success. J. Personal. Social Psychol, 103, 520–542. 10.1037/a0029360 22823287

[B14] Tadmor C. T. Satterstrom P. Jang S. Polzer J. T. (2012b). Beyond individual creativity: the superadditive benefits of multicultural experience for collective creativity in culturally diverse teams. J. Cross-Cult. Psychol. 43, 384–392. 10.1177/0022022111435259

[B15] Vande Berg M. Paige R. M. Connor-Linton J. (2009). The georgetown consortium project: interventions for student learning abroad. Interdis. J. Study Abroad 18, 1–75.

[B16] Walsh R. Walsh M. (2018). In their own words: american students' perspectives on study abroad experiences. Humanist. Psychol. 46, 129–146. 10.1037/hum0000083

